# Preliminary evidence for the acceptability, safety, and efficacy of the flash technique

**DOI:** 10.3389/fpsyt.2023.1273704

**Published:** 2024-01-08

**Authors:** Philip E. Manfield, Graham Taylor, Edie Dornbush, Lewis Engel, Ricky Greenwald

**Affiliations:** ^1^Independent Practice, Albany, CA, United States; ^2^Therapist-Training, Perth, WA, Australia; ^3^Independent Practice, Woodland, CA, United States; ^4^Independent Practice, San Francisco, CA, United States; ^5^Trauma Institute, Northampton, MA, United States

**Keywords:** flash technique, eye movement desensitization and reprocessing, EMDR, subliminal, trauma, painless, flashback, reexperiencing

## Abstract

**Objectives:**

This study reports on four similar studies intended to explore the acceptability, safety, and efficacy of the flash technique (FT), a method of rapidly reducing the intensity of a disturbing memory or image, with minimal subjective disturbance for subjects during the process. Of the four studies, two were conducted during FT trainings in the United States, one in Australia, and one in Uganda.

**Methods:**

The studies involve pre-, post-, and follow-up repeated-measures design to determine the effectiveness of a 15-min FT intervention. A total of 654 subjects were asked to think of a disturbing memory and then participate in a structured experience of an FT. The purpose of this investigation was to determine whether a brief application of an FT would be safe and effective in significantly reducing their disturbance. In each study, subjects rated their disturbing memories on a 0-to-10 scale, with zero representing no disturbance at all and 10 representing the worst they could imagine. Then, they took part in a 15-min group practicum where they were guided in a self-administering FT with no individual supervision or support.

**Results:**

In all four studies, the mean reduction in disturbance exceeded two-thirds, the results were significant (*p* < 0.001), and the effect size was very large. Of the 813 sessions (654 subjects) represented in these studies, only two subjects reported slight increases in disturbances, and both of these subjects reported reductions in disturbance in their second FT experiences 2 h later. At a 4-week follow-up, mean disturbance levels in all four studies indicated maintenance of benefit or slightly further reduction of mean disturbance levels. An 18-month follow-up study with a subgroup of subjects who initially reported a high level of memory-related distress found similar maintenance of gains as well as symptom reduction.

**Conclusion:**

These findings provide preliminary evidence of acceptability, safety, and efficacy of FT; therefore, further study is warranted.

## Introduction

The *Flash Technique* (FT) (1) is a novel easily tolerated variation of exposure therapy that pushes the boundaries on what constitutes exposure to subliminal exposure (2, 3). Various methods have been demonstrated to be effective in treating traumatic memories (4–10). Common to all of these methods is the requirement that clients consciously focus on the trauma memory, generally for a prolonged period, in order to diminish or extinguish subjective disturbance.

While there is a disagreement among researchers on whether a focus on traumatic experiences causes clients to be more likely to prematurely withdraw from treatment (11–15), the requirement of recalling the details of a traumatic event is unappealing to many clients. Studies of client responses to cognitive processing therapy (CPT) and prolonged exposure (PE) at the VA reveal striking numbers of treatment avoidance and premature dropouts and treatment avoidance (16).

FT may provide an alternative to treatments requiring clients to relive their trauma memories. In addition, FT is not burdened by the need for lengthy titration, finding, and processing weakened versions of traumatic memories to make them more tolerable to the client (9), making it more efficient. The four studies reported in this paper are intended to evaluate the safety and effectiveness of FT.

Briefly, the current version of FT (17) involves guiding the client to (a) identify a trauma memory, (b) concentrate on a feel-good engaging activity, relationship, or memory that will serve as a distraction, (c) periodically blink while avoiding thoughts of the trauma memory, and (d) continue to concentrate on the positive engaging distraction. The client during this time is engaging in eye movements or alternating tapping such as what is done with *Eye Movement Desensitization and Reprocessing* (EMDR) (9) but slower. In the context of established broader trauma treatments such as EMDR, CPT, or PE, FT appears to provide an efficient and minimally intrusive therapeutic option for addressing client resistance to those trauma treatments, which require painful exposure (1, 3, 17–22). FT appears to lower clients’ initial disturbance levels while the client maintains a positive focus, making FT relatively well tolerated.

Interventions requiring painful exposure are likely to be better tolerated if the memory being processed becomes less disturbing before those interventions are applied. Many published studies and cases (1, 3, 17–22) suggest that FT is well tolerated by clients. In addition, it is relatively uncomplicated for therapists to learn (23, 24).

FT may represent a significant advance in trauma treatment, but rigorous research is needed. Often, before disseminating a novel intervention with treatment-seeking clients, it is tested on analog subjects. The present four studies were conducted with groups of therapists who were participating in FT workshops.

## Research design

Four similar studies are reported in this study. All studies have pre−/post−/follow-up repeated-measure designs. The first was an FT webinar on 24 March 2018, led by the first and fourth authors (PM and LE); the second was an FT workshop in Atlanta on 6 October 2018, also led by PM and LE; the third was an FT workshop in Sydney, Australia, on 7 February 2019, led by the second author (GT); and the fourth was an FT workshop in Uganda on 3 April 2019, led by the fourth author (ED). The research designs of the four studies were similar with minor variations (see Table 1). The first study included two practicum experiences, each of which had a waiting period of at least 1.5 h between the time that target memories were first identified and the beginning of the practicums. The other three had no waiting period and included only one self-administered practicum experience. The second study had advanced approval from the IRB of Trauma Institute International (approval # 2018–1,001), and data from the other three studies were retroactively included in that approval.

**Table 1:** Summary of differences between the four studies.

Study # (and country)	# subjects (Administered as)	# practical per session	Waiting period	# providing follow-up	% Mean reduction in disturbance at follow-up
1. USA	178 (group)	2	1.5 h	93	66
2. USA	367 (group)	1	NA	98	76
3. Australia	73 (group)	1	NA	54	81
4. Uganda	16 (other)	1	NA	12	87

### Design and method

All four studies used a pre−/post−/follow-up repeated-measures design, in which subjects rated their own subjective memory-related distress prior to a brief FT experience, just after that experience, and 30 days after the FT experience. The question these studies were designed to answer is whether the group application of FT, as it has been implemented in FT presentations, is effective and safe. This question was addressed by analyzing the results of 813 such 15-min guided group applications of FT by 654 subjects with no individual supervision or support. Subjects were also asked to rate their memory-related distress a month later. In one study, an 18-month follow-up was done on the subjects who began with the most distressing memories.

## Study #1

### Subjects

The first and fourth authors (PM and LE) conducted a webinar on 24 March 2018, in which 223 therapists participated. Two 14-min practicums were conducted, in which subjects self-administered FT. Access to two online video demonstrations of FT was offered as an incentive for subjects to provide the results of their practicum experiences. Of the 223 workshop attendees, 178 indicated agreement to participate in the study by sending in their results, thereby yielding information on 353 FT practicum sessions.

### Demographics

All subjects were attending a webinar about FT. As webinar attendees were not required to provide personal data, precise information about the demographics is not available. The subjects were masters- and doctoral-level psychotherapists ranging in the ages of approximately 35–70 years. Approximately 80% of the webinar attendees were women. The subjects were mostly Caucasian, although there was a mixture of races and ethnic origins.

### Exclusion criteria

None of the attendees were excluded from participating in the research. Attendees were told that the technique was designed to avoid emotional pain. However, anyone who believed that they were not psychologically stable enough to participate in the practicum without becoming emotionally upset and labile was discouraged from participating in the practicum experience. The criterion for “too labile” was defined as being likely to become emotional to the extent of not being able to attend to the information being discussed in the workshop or possibly needing counseling or individual attention from a therapist. It is likely that all, or nearly all, subjects engaged in the practicum experience; however, this was not tracked.

### Measures: subjective units of disturbance (SUD) scale

The SUD scale [(25), as adapted by Shapiro (9)] is a simple self-report measure for evaluating the intensity of subjective response to a given disturbing stimulus and, in the present case, for recalling a traumatic memory. The 11-point scale ranges from 0, which is defined as “not disturbing at all,” to 10, which is “the worst you can imagine.” This self-report scale is widely used for evaluating the severity of a traumatic experience and has been shown to correlate with other psychological and autonomic measures of distress (26–29). Non-reactivity to a traumatic memory is considered to be an indicator of recovery from the event (30).

### Procedure: instructions for each practicum

Workshop attendees were given examples of common disturbing memories to assist them in identifying two suitable memories for their practicum experiences. Then, attendees were guided to self-administer FT. A description of the practicum instructions is given below. The full transcript is available upon request from the first author.

#### Choose a disturbing memory

The subjects were directed to identify the disturbing memory or image they wanted to address in the practicum, which was referred to as the “target.” For the purposes of the practicum, they were encouraged to choose one memory or image that had at least a disturbance level of 6 on a scale of 0 to 10 (SUD), was clearly recalled, was not current/ongoing, and did not have a similar earlier memory contributing to its level of disturbance. These limitations were suggested because, although the FT can be used with memories not fitting these guidelines, a memory that meets these guidelines is less likely to require individual attention from a therapist.

Subjects were asked to rate the SUDs that the target memory or image would generate if they let themselves feel the disturbance and to write down that number together with a pair of words that could remind them at a future time of what their target had been. Subjects were discouraged from recalling their disturbing memories vividly. The identification of the target memory is generally done right before beginning FT, but in this webinar (study #1), it was done more than 1 h before FT was initiated. This wait time was intended to control for the possibility that just thinking of the memory might cause a reduction in disturbance that could be mistaken for an effect from FT.

#### Positive engaging focus

Subjects were asked to focus on imagining an activity, animal, person, memory, or music selection that provided an immediate experience of pleasure or was at least positive and engaging, for which examples are provided below.

#### Tap your thighs

The subjects were directed to alternately tap one thigh and then the other: “I am going to tap my thighs, and I’d like you to copy my movements, tapping your thighs while focusing on the positive engaging activity, memory, or person you have just thought of. Thinking of this will give you an alternative focus as a substitute for the disturbing memory.”

#### Flash

In two successive sets of slow tapping, subjects were asked to focus on the PEF. In the first short set, subjects were simply asked to tap and focus on the PEF without thinking of the disturbing material in order to see whether they could do that. In the second set, subjects were to tap, focus on the PEF, and then blink once when the leader said the word “flash.” This second set gave subjects an opportunity to practice maintaining their focus on the PEF while both tapping and blinking.

#### A total of four sets of triple blinks

After completing this preparation, subjects were instructed to focus on the PEF, continue tapping slowly, and perform a set of three rapid eye blinks when the leader said the word “flash.” Approximately every 7 s, the leader repeated the word “flash” and subjects were to rapidly blink their eyes three times. After five of these triple blinks, subjects were asked to stop tapping and blinking and, without thinking of the target memory intensely, to notice any change that may have occurred in it. The sequence—including tapping, five sets of triple blinks, and checking for a change in the target memory—was repeated three more times for a total of four sets.

#### Data collection

At the end of the fourth repetition of five triple blinks, subjects were asked to write down the final SUD levels on the response sheets, where the initial SUD levels and reminder words had been recorded, and to indicate their willingness to be research subjects by handing them in at the end of the workshop. For study #1, a webinar which involved handing the response sheets in constituted filling out a web-based survey containing the results they had written down, as well as their reminder words and email addresses.

### Results

A total of 353 sessions were reported by 178 out of a total of 223 subjects. A *generalized linear model* (GLM) repeated-measures ANOVA found a significant mean reduction pre-FT to post-FT for nearly two-thirds of the workshop attendees, from 6.80 (*SD* = 1.379) to 2.35 (*SD* = 1.888), *F*(1, 352) = 1626.578, *p* < 0.001, *η*^2^ = 0.822 (as in all GLM performed for this study, a sphericity assumption violation was anticipated, so a Greenhouse–Geisser adjustment was performed of the degrees of freedom). A small increase in disturbance (SUD) was reported in only 2 out of the 353 sessions. Both subjects who reported these increases subsequently reported a reduction in disturbance in their second practicum experience 2 h later.

#### The group providing follow-up was representative

More than half of the subjects (93 of 178) provided follow-up data for 183 sessions. To assess whether the group of 93 attendees who provided follow-up information was representative of the group of 85 attendees who had not, the change in the SUD score of subjects pre- to post-FT for the two groups was evaluated using a one-way ANOVA. The null hypothesis for this comparison was that the two groups were not significantly different from each other, which was confirmed by the analysis. No significant differences were observed between pre-treatment (*M* = 4.467, *SD* = 2.024) and post-treatment (*M* = 4.213, *SD* = 2.113) SUD changes in the groups, *F*(1, 374) = 1.334, *p* = 0.249, *η*^2^ = 0.004. This supports the representativeness of the data obtained from the follow-up.

#### Main effect

**Figure 1:**
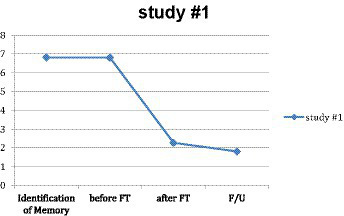
Main effect in study #1.

For the 183 sessions where all four measures were available, a GLM repeated-measures ANOVA was performed to test the significance and effect size of the main treatment result. There was a significant reduction in mean SUD levels for this subgroup from pre-treatment (*M* = 6.85, *SD* = 1.309) to post-treatment (*M* = 2.27, *SD* = 1.834), *F*(1, 183) = 949.335, *p* < 0.001, *η*^2^ = 0.838 (see Figure 1). The reduction was similarly significant for all subjects from pre-treatment (*M* = 6.80, *SD* = 1.379) to post-treatment (*M* = 2.35, *SD* = 1.888), *F*(1, 352) = 1626.578, *p* < 0.001, *η*^2^ = 0.822. A rule of thumb for effect size is that partial eta squared (in this case, the same as eta squared) is considered large—and clinically significant—if it is above 0.14 (31). Moreover, 0.838 and 0.822 represent very large effect sizes. Notably, more than a third of the subjects (130 out of 353) reported SUDs of 0 or 1 at the end of the 14-min practicum.

#### Effect of the waiting period

The differences in the mean SUD levels between the beginning of the waiting period when the target memory was first identified and the beginning of the treatment phase reflected a lowering of SUD levels. A repeated-measures ANOVA determined that this reduction was not significant (*M* = 6.81, *SD* = 1.394) (*M* = 6.80, *SD* = 1.379), *F*(1, 352) = 0.438, *p* = 0.509, *η*^2^ = 0.001. This indicates that just identifying the disturbing memories to be the focus of FT and evaluating their level of disturbance did not result in a significant change in SUD levels.

Additionally, a significant decrease in mean SUD levels was observed from *M* = 2.27 (*SD* = 1.834) at post-treatment to *M* = 1.81 (*SD* = 1.733) at follow-up, *F*(1, 182) = 12.411, *p* = 0.001, *η*^2^ = 0.062, indicating that, for some subjects, an additional reduction in disturbance occurred after the treatment phase ended. The effect size was medium.

## Study #2

### Subjects

The first and fourth authors (PM and LE) conducted a 1.5-h workshop about FT at the 2018 Annual Conference of the EMDR International Association in Atlanta, Georgia. All workshop subjects were invited to take part in a short practicum and asked to indicate their willingness to participate in the research study by turning in their response sheets, on which they had written their beginning and ending disturbance (SUD) levels during the practicum and their email addresses. Access to two online video demonstration recordings of FT was offered as an incentive for participation.

A total of 473 clinicians had enrolled in the 1.5-h workshop about FT, which included the 14-min practicum component. Of those clinicians, 367 agreed to have their results used in this research. Demographics, exclusion criteria, and practicum instructions were similar to those in study #1.

### Results

#### Main effect

Three hundred and sixty-nine subjects handed in their response sheets. A repeated-measures ANOVA found a significant mean SUD reduction pre-FT (*M* = 2.317, *SD* = 1.682) to post-FT (*M* = 7.395, *SD* = 1.507) of more than two-thirds of the subjects, *F*(1, 366) = 2716.133, *p* < 0.001, *η*^2^ = 0.881. For this study, the practicum resulted in a complete resolution for a third of the subjects (122 out of 367), who reported SUDs of 0 or 1 at the end of the 14-min practicum.

#### Follow-up

Ninety subjects could not be contacted for the follow-up, because the email addresses they submitted were illegible, which was assumed to be a random factor. A total of 98 out of the 277 contacted subjects provided follow-up data about how their results had held up after 4 weeks. To assess whether the 98 subjects who provided follow-up information were representative of the group (*N* = 179) who had not, the change in the SUD score of subjects pre- to post-FT for the two groups was evaluated using a one-way ANOVA. As in the analysis for study #1, no significant difference was found between pre- and post-treatment SUD change in the two groups (*M* = 5.06, *SD* = 1.930) (*M* = 5.12, *SD* = 1.713), *F*(1, 366) = 0.091, *p* = 0.763, *η*^2^ = 0.000. This supports the representativeness of data obtained from the follow-up.

#### Subjects who completed the study

**Figure 2:**
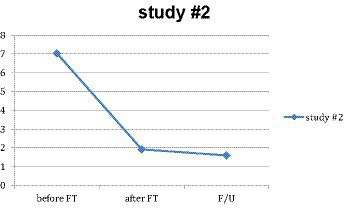
Main effect in study #2.

For the 98 subjects who provided all three measures, a repeated-measures ANOVA was done to test the significance and effect size of the main treatment result pre- and post-treatment for that subgroup. Similarly, the main effect within subjects was highly significant, and the effect size was very large (*M* = 7.028, *SD* = 1.427) (*M* = 1.680, *SD* = 1.877), *F*(1, 89) = 441.459, *p* < 0.001, *η*^2^ = 0.832 (see Figure 2).

An insignificant decrease in mean SUD levels was observed from *M* = 1.916 (*SD* = 1.481) at post-treatment to *M* = 1.60 (*SD* = 1.832) at follow-up, *F*(1, 97) = 2.966, *p* = 0.088, *η*^2^ = 0.030.

For the 98 subjects who completed all measures, the reduction during the entire study period, from pre-FT intervention to follow-up, was highly significant with a very large effect size (*M* = 7.046, *SD* = 1.418) (*M* = 1.60, *SD* = 1.832), *F*(1, 97) = 595.672, *p* < 0.001, *η*^2^ = 0.860.

#### Highly distressed subjects

A total of 36 subjects chose memories for the FT practicum that they rated 10 (the worst they could imagine) and 40 others chose memories that they rated as 9 (nearly the worst they could imagine.) To further evaluate the safety of FT for people with extremely high levels of disturbance, a separate GLM repeated-measures ANOVA was performed on the data from the 75 subjects who had begun with a disturbance level (SUDs) of 9 or 10. The mean reduction in SUD scores from *M* = 9.480 (*SD* = 0.503) at pre-FT to *M* = 2.847 (*SD* = 1.930) at post-FT was 70% and highly significant, *F*(1, 74) = 844.517, *p* < 0.001, *η*^2^ = 0.919. The effect size was very large. Of these subjects, none experienced an increase but only one did not experience a reduction of disturbance.

#### Follow-up of highly distressed subjects

Although not originally part of the above study design, follow-up emails were sent to these 75 subjects 18 months after the 90-min presentation they participated in. They were asked to fill out an anonymous online survey in return for which they would be given access to two current video recordings of FT demonstrations. A total of 23 subjects, or just over 30%, responded. In the follow-up request, subjects were supplied with the two words they had originally designated as reminders of their target memory and asked 10 questions including the current level of their disturbance after a year and a half.

One respondent could not remember what his or her target memory was. During the original practicum experience, the mean disturbance level for 75 subjects in this group had reduced from 9.480 to 2.8. The mean disturbance level for the 22 subjects responding to the follow-up survey after 18 months was slightly lower (2.3), with three reporting current disturbance levels of four and five, but all other current disturbance levels were lower. One participant had subsequently received additional treatment for his or her issue. Excluding this participant, the mean disturbance level reported was slightly lower (2.2). The average time between the practicum and the occurrence of respondents’ disturbing memories was more than 8 years. After the practicum experience, 17 of the 23, or 74%, subjects indicated that they had experienced symptom reduction. As therapists, the responding subjects had conducted a combined 791 sessions involving FT since receiving 90 min of instruction in FT. FT was reported as helpful to the treatment in 83% of those sessions and had not been found to be harmful in any.

## Study #3

### Design

The second author (GT) conducted a series of three brief trainings on FT in Sydney, Australia, from 18 November 2018 to 19 January 2019 as part of three accredited EMDR trainings. The procedure in all three trainings was identical, and the data from the 73 therapists involved were combined into one study.

### Subjects, demographics, and procedures

Demographics, exclusion criteria, data collection, and other procedural details including instructions for performing the self-administered practicum were essentially the same as those used in study #2, with the exception that in Australia, where 85% of the subjects were women and 97% of the subjects were Caucasian. The subjects were free to select what memory to use in the practicum, and, for those who did not readily think of another disturbing memory, a shame-related memory was suggested because shame memories tend to be plentiful in most people’s histories.

### Results

All 73 workshop attendees provided their pre- and post-FT SUD ratings. A GLM repeated-measures ANOVA found a significant mean reduction of over 80% (*M* = 7.719, *SD* = 0.954) (*M* = 1.260, *SD* = 1.675), *F*(1, 72) = 830.944, *p* < 0.0001, *η*^2^ = 0.920. Moreover, no subjects reported an increase in disturbance.

#### The group providing follow-up was representative

For this study, more than half the subjects (54 of 73) provided follow-up data. To assess whether the data reported by those 54 subjects were representative of the data reported by the group of 19 who did not provide a follow-up, the change in SUDS pre- to post-treatment was evaluated with a one-way ANOVA to determine whether there was an interaction between the two groups. There was no significant interaction between the changes in SUD reported by the two groups (*M* = 8.194, *SD* = 12.741) for the group providing follow-up and (*M* = 6.737, *SD* = 0.991) for the group that did not provide follow-up, *F*(1, 53) = 0.246, *p* = 0.622, *η*^2^ = 0.003, indicating that no significant difference between mean pre- and post-treatment SUD change for the 54 subjects for whom follow-up data were available and the 19 subjects for whom the follow-up data were unavailable.

#### Main effect

**Figure 3:**
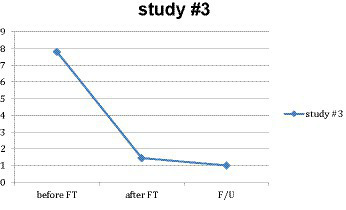
Main effect in study #3.

For the 54 subjects for whom all three measures were available, a repeated-measures ANOVA was performed to test the significance and effect size of the main treatment result. There was a significant reduction in mean SUD levels for this subgroup from *M* = 7.806 (*SD* = 1.039) at pre-treatment to *M* = 1.444 (*SD* = 1.870) at post-treatment, *F*(1, 53) = 505.181, *p* < 0.001, *η*^2^ = 0.905 (see Figure 3); where 0.905 partial eta squared represents a very large effect size. There was a further significant reduction at a 4-week follow-up (*M* = 1.000, *SD* = 1.727), *F*(1, 53) = 1.229, *p* < 0.001, *η*^2^ = 0.314. For this study, the practicum resulted in a complete resolution for more than two-thirds of the subjects (52 out of 73), who reported SUDs of 0 or 1 at the end of the 14-min practicum.

#### Highly distressed subjects

Two subjects chose memories for the FT practicum that they rated 10 and 11 (the worst they could imagine) and others rated their memories 9 (nearly the worst they could imagine). To further evaluate the safety of FT for people with extremely high levels of disturbance, a separate repeated-measures ANOVA was performed on the data from the 13 subjects out of 73 who had begun with a disturbance level (SUDs) of 9 or 10 out of 10. The mean reduction in disturbance pre- to post-FT was more than two-thirds and very significant (*M* = 9.154, *SD* = 0.104) (*M* = 2.077, *SD* = 0.702), *F*(1, 12) = 96.548, *p* < 0.001, *η*^2^ = 0.889. The effect size was very large. Of these subjects, none experienced an increase, and only one (1%) did not experience a reduction in disturbance.

## Study #4

### Design

The third author (ED) conducted a small 5-h training in advanced clinical skills for EMDR therapists in Uganda on 3 April 2019, which included a brief training in FT. Fifteen therapists participated in the training and took part in two practicum experiences of less than 15 min each. The first practicum replicated the procedure used in studies #1 through #3, while the second involved subjects performing FT on each other.

### Subjects, demographics, and procedures

The demographics, exclusion criteria, data collection, and other procedural details including instructions for performing the self-administered practicum were essentially the same as those used in study #2, with the exception that conducted in Uganda, where all subjects were people of color. One participant was late for the first practicum, and her data were only included in the data from the second practicum. Thirteen of 16 subjects were women (81%).

### Results

#### Main effect

For the guided practicum, 15 subjects provided their pre- and post-FT SUDs ratings. A repeated-measures ANOVA was performed to test the significance and effect size of the main treatment result. There was a significant mean reduction from *M* = 8.20 (*SD* = 1.265) pre-FT to *M* = 1.20 (*SD* = 1.521) post-FT of over 80%, *F*(1, 14) = 245.0, *p* < 0.0001, *η*^2^ = 0.946. All but two subjects reported SUD decreases of six or greater. The remaining two subjects reported SUD decreases of three and four.

#### Follow-up

**Figure 4:**
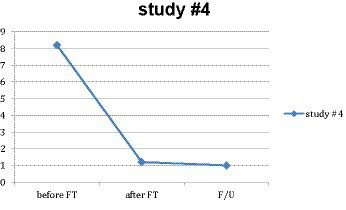
Main effect in study #4.

Twelve subjects provided a 4-week follow-up. The mean reduction for these 12 people from *M* = 8.25 (*SD* = 1.422) pre-FT to *M* = 1.0 (*SD* = 1.348) at the follow-up was 7.25 (87%) (see Figure 4). These results were significant to *p* < 0.001, *F*(1, 20) = 98.807, *η*^2^ = 0.900. Whether the 12 subjects who provided the follow-up were representative of the 15 who did not could not be verified mainly due to the small number of subjects (Table 2).

**Table 2:** Main effects for completers (four studies).

Study #	# of subjects	Measure #1 (before FT)		Measure #2 (after FT)		Measure #3 (30 day F/U)	
1	93	*M* = 6.85	*SD* = 1.309	*M* = 2.27	*SD* = 1.834	*M* = 1.81	*SD* = 1.733
2	98	*M* = 7.028	*SD* = 1.427	*M* = 1.680	*SD* = 1.877	*M* = 1.60	*SD* = 1.832
3	54	*M* = 7.806	*SD* = 1.039	*M* = 1.444	*SD* = 1.870	*M* = 1.0	*SD* = 1.727
4	12	*M* = 8.25	*SD* = 1.422	*M* = 1.0	*SD* = 1.348	*M* = 1.0	*SD* = 1.348

Hedges g values for the four studies are 2.9, 3.2, 4.2, and 5.2, respectively.

## Discussion

### Safe and effective

**Figure 5:**
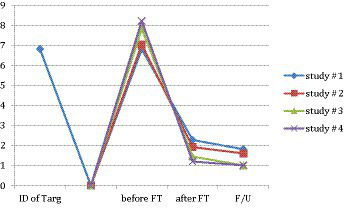
Comparison of all four studies.

In study #1, 178 professionals self-administered FT twice each and achieved a mean SUD reduction in disturbance levels pre-FT from *M* = 6.80 (*SD* = 1.379) to post-FT M = 2.35 (*SD* = 1.888) of nearly two-thirds, in less than 15 min. In study #2, 367 professionals self-administered FT and achieved a mean SUD reduction of over two-thirds, from *M* = 7.395 (*SD* = 1.5066) to *M* = 2.317 (*SD* = 1.6802) in less than 15 min. Moreover, less than 1% (3 out of 367) of subjects failed to experience a reduction in their distress, and none of the 367 subjects reported an increase in their disturbance levels. In study #3, 73 professionals self-administered FT and achieved a mean SUD reduction pre- to post-FT of more than 80%, from *M* = 7.719 (*SD* = 0.954) to *M* = 1.260 (*SD* = 1.675) in less than 15 min. In study #4, 15 professionals self-administered FT and achieved a mean SUD reduction pre- to post-FT of more than 80%, from *M* = 8.20 (*SD* = 1.265) to *M* = 1.20 (*SD* = 1.521) in less than 15 min. The results from all four studies were very similar (see Figure 5). Of the four studies, only two sessions of FT out of 813 (654 subjects) resulted in an increase in disturbance (study #1). These two subjects, however, both experienced a disturbance reduction in the second of their two practicums. These studies provide preliminary evidence for the efficacy and safety of FT.

### Easy to learn

FT can be rapidly learned by clinicians. Over 6,000 clinicians have attended a 6-h webinar in FT over the past 28 months, of which over 1,500 have joined a listserv devoted to discussing innovations and issues about the FT. Follow-up data on their results are not available. As stated earlier, however, we do have data from 23 of the therapists from study #2 who received 1.5 h instruction in FT. Nineteen out of 23 of these clinicians reported using FT with clients a total of 791 times and found it helpful in 83% of those instances.

## Limitations and strengths

Limitations of these studies include the lack of a control group, inability to control for possible expectancy effects, the use of a single outcome measure which is subjective, and the use of a non-clinical convenience sample rather than a clinical population. For the 18-month follow-up, the low response rate is an additional limitation. In addition, the measure used in these studies considered only the level of the subjects’ perceived disturbance and did not measure PTSD symptom change.

The purpose of preliminary studies with non-treatment-seeking participants is to ascertain whether the treatment is sufficiently promising so as to warrant further study. FT is indeed sufficiently promising, and earlier conference presentations of these findings have inspired the next steps. Further research should include randomized comparisons of FT to other legitimate trauma treatments, tracking not only effectiveness but also efficiency and dropout rate, ultimately with treatment-seeking subjects. The planned RCT by Babaei and Ritvo (23) is an RCT expected to present such a comparison. The RCT by Brouwers et al. (18) compared the mechanism of EMDR to that of FT; however, it was limited to 8-min treatment sessions with non-treatment-seeking subjects. It showed 8 min of FT to be as effective as 8 min of EMDR in symptom reduction and better tolerated. A more robust study with one, and preferably multiple, full-length session with a treatment-seeking population is required to allow both techniques to demonstrate their full capability. Beyond the reduction of disturbance, additional research is necessary to establish whether FT can both reduce trauma-related disturbance and produce stable symptom relief.

A small randomized controlled study (3) demonstrated the effect of three group sessions of FT on PTSD resulting from traffic accidents. Other studies indicated the effectiveness of FT in groups (3, 7, 20–22). More research is recommended to evaluate the potential of FT, as used in Study #1 and Study #2, in providing low-cost relief to large groups suffering from natural or man-made disasters.

Strengths of the studies in this article include a large number of subjects, the use of a valid and reliable outcome measure, the waiting period in study #1, and the use of a scripted, replicable intervention. The 18-month follow-up for study #2, of only those subjects who initially reported the highest level of memory-related distress, indicates that FT may work not only with minor memories but also with significant trauma memories, consistent with other studies and case reports (3, 17, 19–22). The 18-month follow-up also further supports the stability over time of FT results. In addition, studies #3 and #4 were conducted by unaffiliated researchers on other continents and achieved results similar to those of studies #1 and #2 conducted by the first and fourth authors in the USA, suggesting that these results are robust and replicable. The waiting period in study #1 controlled for attention and the passage of time. Because the scripted intervention was applied in a large group, rather than being individualized, the present findings may underestimate the benefit that would be expected when FT is done by a clinician with an individual client. This possibility is supported by study #4 obtaining the largest mean reduction in disturbance (87%); study #4 was the only study in which FT instructions were administered by subjects with each other.

### Qualitative feedback from the subjects

FT has been widely accepted by clinicians and integrated into their trauma treatment approaches. Since these studies were initiated, over 12,000 professionals have been trained in FT, mostly via webinars like the one on which study #1 is based. Over 4,700 have joined an active FT listserv on which the descriptions of clinical FT successes are reported daily and refinements are often proposed. Subjective responses collected by the sponsoring organization from the subjects in study #2, the EMDR International Association, also indicated a high level of acceptance of FT. In total, 97% of subjects indicated that this material was of personal value to them, and 91% of those responding indicated that they thought FT was likely to be useful in their clinical practices (Manfield, 2020, Unpublished data).

### Safety of FT

The safety of FT is indicated by the absence of adverse outcomes in any of these four studies included in this study. In study #2, the largest of these studies, no participant reported an increase in the SUD level from pre- to post-FT, and less than 1% (3 out of 367) reported a lack of reduction in disturbance. In the 18-month follow-up of the 75 members of this study group with initial SUD levels of 9 or 10, respondents, all clinicians, reported having used FT collectively in a total of 791 sessions with no harm caused to any of those clients. This is consistent with published cases in which FT was safely used with clinical populations (1, 3, 17–22).

### Proposed mechanism of action

We believe that FT inhibits the reexperiencing of historical trauma by distracting clients, allowing for more rapid and effective present-day processing of the trauma. Most clients with PTSD reexperience aspects of their traumatic memories when instructed to visualize the image or memory (32, 33). This reexperiencing may reinforce their disturbing associations with the traumatic images or memories. One explanation of the effectiveness of FT may be that it prevents reexperiencing traumatic memories by limiting subjective awareness or conscious accessing of those memories. Particularly with fear, the conscious response to those memories inhibits the activity of the prefrontal cortex (2, 34), making mental processing of the memories less effective. Without conscious accessing of the traumatic memory, defenses used to prevent experiencing painful emotions associated with the memory are not activated (2, 35). Referring to the areas of the brain that process fear, Siegel reports that fMRI demonstrated that “Conscious exposure suppressed activity in these regions and did not diminish fear.”

### Working memory

Using FT requires concentrated attention on an “engaging focus.” One cannot rule out the possible effect of the tax on working memory contributing to the disturbing memory becoming less intense. It is possible that clients may be unable to maintain an intense focus on a positive memory and simultaneously retain a vivid and disturbing memory of the trauma (9, 36). It seems unlikely, however, that taxing working memory alone can account for the unusually rapid and effective nature of FT demonstrated in this study, which suggests that FT may have a different or additional mechanism of action. Moreover, the bilateral stimulation administered in FT is so slow that, although it can cause a relaxation response (37, 38), it is doubtful whether it taxes working memory enough to be solely responsible for the large impact that FT produces. Francine Shapiro’s adaptive information processing model (AIP) posits that the brain automatically works to resolve traumatic memories (9). Adding what Siegel has shown with fear, this occurs most effectively when the parts of the brain necessary for this processing are not inhibited by being consciously upset.

### Memory reconsolidation

Ecker et al. (4) and Lee (39) proposed that, for effective memory reconsolidation, there must be a “prediction error,” which signals to the brain that the memory may need modification because it is not accurate enough to ensure future safety. FT appears to produce several possible prediction errors, including the absence of affect and emotion during the accessing of the memory and relaxation response because of physiological effects of slow eye movements (37) (Schubert et al., 2011); a sense of reduction in intensity when the memory is reassessed between sets of five triple blinks; observer position and mindful stance as opposed to reexperiencing, providing a sense of distance and acceptance; and inhibition of conscious defense mechanisms, preventing potential dissociation, avoidance, or abreaction that would typically be present when recalling the memory.

### Subliminal processing of trauma

During FT, clients think of a distraction (PEF) and generally do not report thoughts of their original disturbance. Their connection to the disturbing memory is subliminal in that they are, in the moment, consciously unaware of thinking of the disturbance. However, the memory is nevertheless a focus of painless unconscious mental activity (40). The human brain engages in a high level of activity outside of conscious awareness, including when a person appears to be at rest (41). Processing of fear can occur even when the person is not consciously aware of what the brain is doing, and this processing can nevertheless impact emotional responses, attitudes, and behaviors (42–44). Siegel states in his fMRI study of spider phobia, “Overall, we believe our findings establish a neurobiological basis for the effects of non-conscious exposure, indicating recruitment of brain regions that support automatic fear extinction.” Importantly, the same stimuli or messages delivered in such a way that they could be consciously perceived have been shown to be significantly *less* effective in reducing fear than when they were “unreportable” (34, 35, 42). Although Siegel et al. and Taschereau-Dumouchel et al. have focused their research on phobias, both suggest in their articles that the results should be generalizable to other conditions. We believe that Siegel’s results do generalize to the trauma conditions we process with FT and explain to a large extent the reduction in disturbance produced by FT.

Siegel’s functional magnetic resonance imaging (fMRI) study (2012) demonstrates that parts of the prefrontal cortex that play an important role in processing fear are active when the subject is not aware of a “Fight or Flight” reaction and become relatively inactive when a subject is aware of such a reaction. Although no fMRI studies have been completed about the FT, the technique is designed to prevent such a reaction, and it is apparent that subjects are not aware of such a reaction during the administration of the technique.

We believe that the triple blinks serve to subliminally bring to mind the disturbing content that is the focus of the process. In his theoretical article, Wong (45) points out that, during FT, the PEF is key in preventing clients from being overwhelmed or even activated by the disturbing memory being processed. He posits that blinking momentarily interrupts that role, allowing the disturbance to momentarily emerge and be recognized and processed subliminally. Because the amygdala takes a longer time to react to disturbing stimulation, it does not have sufficient time to react during those moments, so clients do not consciously recognize the disturbance. In this way, subliminal processing continues to occur without the client’s conscious awareness.

## Conclusion

FT has shown promise as a way to prepare clients for their challenging trauma therapy work and to accelerate that work. It is easily mastered by therapists, and it appears to be safe, rapid, effective, and well tolerated by clients. Consistent with prior publications of FT with clinical cases, the present findings suggest that FT is safe, impactful, and worthy of further research.

## Clinical impact statement

The flash technique (FT) is a recently developed procedure for rapidly reducing the intensity of disturbing memories or images, with minimal subjective disturbance for clients during the process. A mean reduction in disturbance of more than two-thirds after 15 min of group-administered FT is reported for each of four studies totaling 813 sessions (654 subjects), maintained at follow-up and with no adverse impact on any participant. The method used in these studies may be highly scalable. If these findings are replicated with clinical populations, FT may make trauma-focused psychotherapy quicker and better tolerated. These studies involved a sample of convenience and no control and must be replicated in randomized controlled studies with clinical populations.

## Data availability statement

The original contributions presented in the study are included in the article/supplementary material, further inquiries can be directed to the corresponding author.

## Ethics statement

The studies involving humans were approved by the Trauma Institute’s IRB approval # 2018-1001. The studies were conducted in accordance with the local legislation and institutional requirements. The participants provided their written informed consent to participate in this study.

## Author contributions

PM: Conceptualization, Formal analysis, Investigation, Methodology, Project administration, Writing – original draft, Writing – review & editing. GT: Investigation, Project administration, Writing – original draft. ED: Investigation, Project administration, Writing – original draft. LE: Conceptualization, Investigation, Methodology, Project administration, Writing – original draft, Writing – review & editing. RG: Conceptualization, Resources, Writing – original draft, Writing – review & editing.

## Appendix

The data reported in this manuscript have not been previously published in any other work. All data were collected specifically for the purpose of publication in this manuscript. No other manuscripts incorporate any of the four datasets upon which this manuscript is based.
